# Supplementary material for: Three-dimensional total-internal reflection fluorescence nanoscopy with nanometric axial resolution by photometric localization of single molecules
Source: Nat Commun. 2021 Jan 22;12:517. doi: 10.1038/s41467-020-20863-0 (PMC7822951; doi:10.1038/s41467-020-20863-0)
Supplement: Supplementary file 1 — Supplementary Information [file 41467_2020_20863_MOESM1_ESM.pdf]

Supplementary Information

Three-dimensional total-internal reflection fluorescence nanoscopy with nanometric axial resolution by photometric localization of single molecules

Alan M. Szalai, Bruno Siarry, Jerónimo Lukin, David J. Williamson, Nicolás Unsain, Alfredo Cáceres, Mauricio Pilo-Pais, Guillermo Acuna, Damián Refojo, Dylan M. Owen, Sabrina Simoncelli and Fernando D. Stefani

**Supplementary Figure 1**	Quantification of the axial mislocalization (Δz) when using the exponential approximation or incorrect calibration parameters θ_i, α and N_0
**Supplementary Figure 2**	SIMPLER protocol workflow.
**Supplementary Figure 3**	Example photon count histograms of a DNA-PAINT experiment, before and after frame filtering.
**Supplementary Figure 4**	Calibration of the TIRF excitation angle.
**Supplementary Figure 5**	Example percentual axial distortions introduced when using incorrect calibration parameters.
**Supplementary Figure 6**	Examples of SIMPLER reconstructions using different computation methods and varying θ_i, α and N_0.
**Supplementary Figure 7**	Screenshot of the Supplementary Software for *angle and alpha tuning* operation output.
**Supplementary Figure 8**	Influence of the first and last frame filtering step on image quality.
**Supplementary Figure 9**	Microtubules from hippocampal neurons immunolabeled for DNA-PAINT super-resolved in 3D using SIMPLER in a custom-made setup
**Supplementary Figure 10**	Microtubules from Human Fetal Foreskin Fibroblasts cells, immunolabeled for DNA-PAINT super-resolved in 3D using SIMPLER using a commercial setup (Nikon STORM 5.0).
**Supplementary Figure 11**	Image decorrelation analysis.
**Supplementary Figure 12**	Field-dependent z-uncertainty
**Supplementary Figure 13**	Microtubules immunolabeled for dSTORM super-resolved in 3D using SIMPLER
**Supplementary Table 1**	Axial dependence of the collected fluorescence signal.
**Supplementary Table 2**	Effect of calibration parameters for axial determination via SIMPLER.
**Supplementary Table 3**	DNA docking and imager strands sequences.

Supplementary Figures

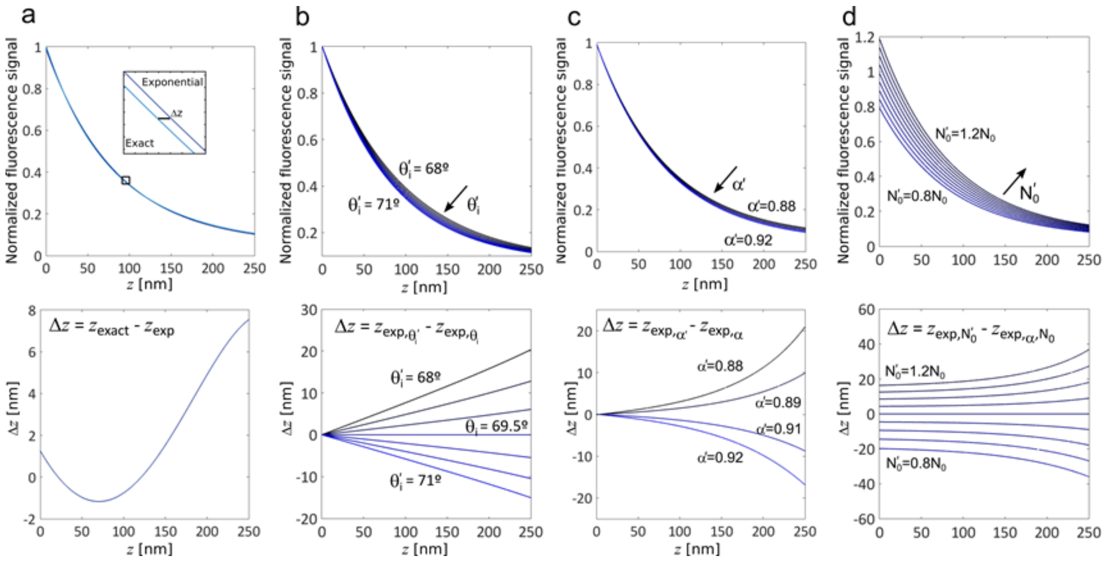

Supplementary Figure 1. Quantification of the axial mislocalization (Δz) when using the exponential approximation, or incorrect calibration parameters $\boldsymbol{\theta_i}$, $\boldsymbol{\alpha}$ and $\boldsymbol{N_0}$ (**a**) Exact solution and exponential fit of $F(z)$ for the experimental conditions of our experiments (θ_{i} = 69.5°, α = 0.90). Inset: Δz between the curves for the range of z from 71 to 78 nm. Bottom: Δz between the exact solution and the exponential approximation as a function of z. For z <200 nm, Δz < 6 nm. In the range of 0-150 nm, Δz <1 nm. (**b-d**) Exponential $F(z)$ (top) and Δz (bottom) as a function of z, for ranges of θ_{i} , α and N_0 around the correct experimental values. (**b**) θ_{i} = {68°, 68.5°, 69°, 69.5°, 70°, 70.5°, 71°}. An incorrect θ_{i} by $\pm$1.0° leads to Δz < 7 nm for $z < 150$ nm, and Δz < 13 nm for $z < 250$ nm. (**c**) α = {0.88, 0.89, 0.90, 0.91, 0.92}; i.e. a range corresponding to a non-evanescent illumination component $(1-\alpha)$ of 12% and 8% of the total power at $z = 0$. A 10% incorrect α generates Δz < 10 nm in all the range from 0-250 nm. (**d**) N_0= {0.8, 0.85, 0.90, 0.95, 1.0, 1.05, 1.10, 1.15, 1.20}N_0. An overestimation of N_0 by 10%, leads to Δz = 8.5 nm at z = 0 and Δz = 18.1 nm at z = 250 nm.

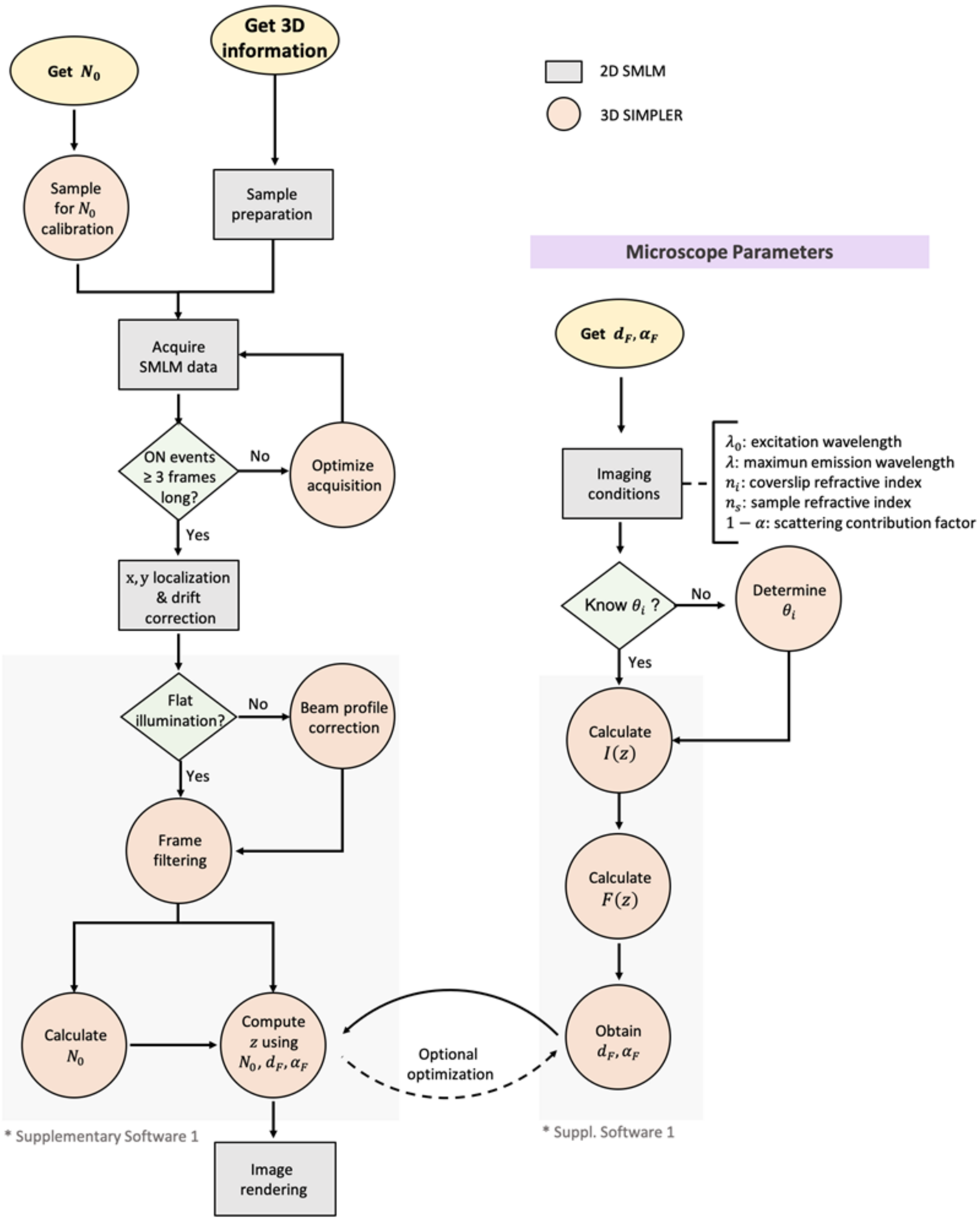

Supplementary Figure 2. SIMPLER protocol workflow. Starting with sample preparation, the user should prepare an additional sample to calibrate N_0 for the same imaging conditions as for the biological experiments. One option to prepare such a sample is to simply deposit the same fluorescent label used for biological imaging on a coverslip. Next, data acquisition is performed as in any typical 2D SMLM method with the caveat of adjusting the acquisition/experimental conditions (power, frame rate, dSTORM switching buffer, DNA imager/docking sequence pair) so that the average single molecule emission event last at least three camera frames. During image analysis, single-molecule fluorescence events are localized, drift-correction procedures are applied and photon counts can be corrected for uneven illumination. Then, special emphasis is given to filtering the localization list in order to exclude the first and last frames of each single-molecule emission event. To convert the number of emitted photons to z-positions, three parameters are needed: N_0, d_F and α_F. N_0 is obtained by data analysis of the calibration sample. d_F and α_F are obtained from a fit to: $F(z) =$

$I(z) \times \mathrm{CF}_{\mathrm{avg}}(z)$, which is defined by the microscope set-up and sample/imaging conditions. The only experimental requirement to estimate these two parameters is to determine the angle of incidence of the excitation light θ_i. While some commercial set-ups already provide this value, a simple option to do this is to use the displacement method as described in Supplementary Method 1. SIMPLER is quite robust against mistaken values α (see Supplementary Figures 1, 5 and 6). To simplify the adoption of SIMPLER, we provide Supplementary Software 1 and example data. The software also permits the adjustment of the calibration parameters using images of reference structures, such as microtubule cross-sections or nuclear pore complexes.

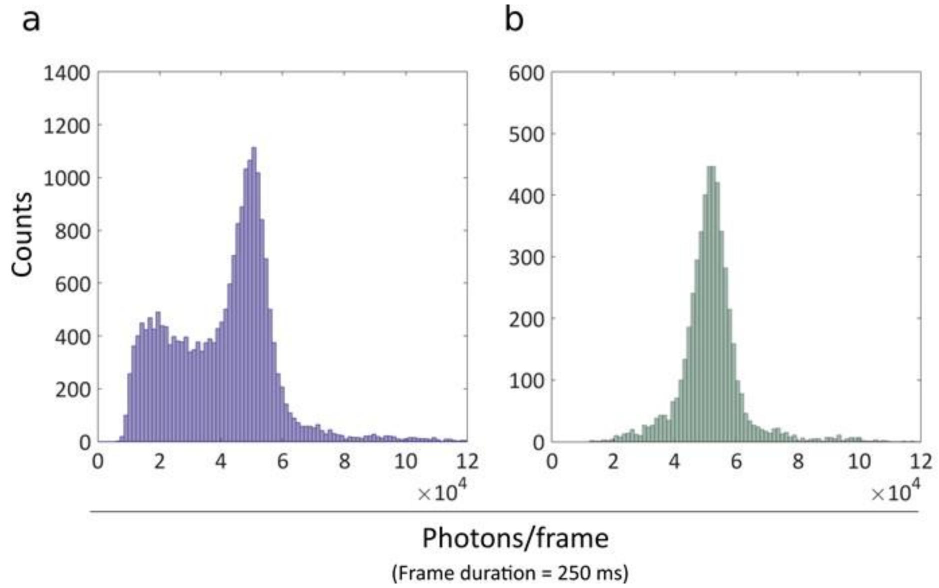

Supplementary Figure 3. Example photon count histograms of a DNA-PAINT experiment, before and after frame filtering. (a) Histogram of photon counts per frame (frame time 250 ms) for all localizations detected in the sample for the determination of $\widehat{N_0}$ (total localizations: 21,639). (**b**) Histogram of photon counts per frame for the valid localizations after frame filtering (total valid localizations: 5,030). The frame filtering procedure eliminates low count frames originated from i) specific DNA binding events that lasted less than three frames, and ii) shorter events due to non-specific binding.

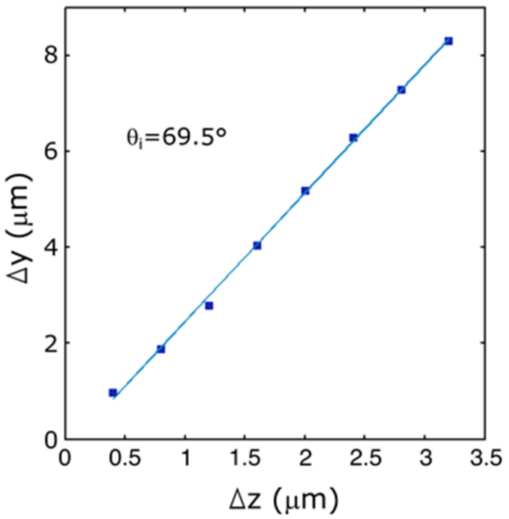

Supplementary Figure 4. Calibration of the TIRF excitation angle. Displacement of the excitation laser beam center Δy versus the axial position of the sample Δz (according to Supplementary Method 1). Linear fitting allows determination of θ_i = 69. 5º ± 0.7º.

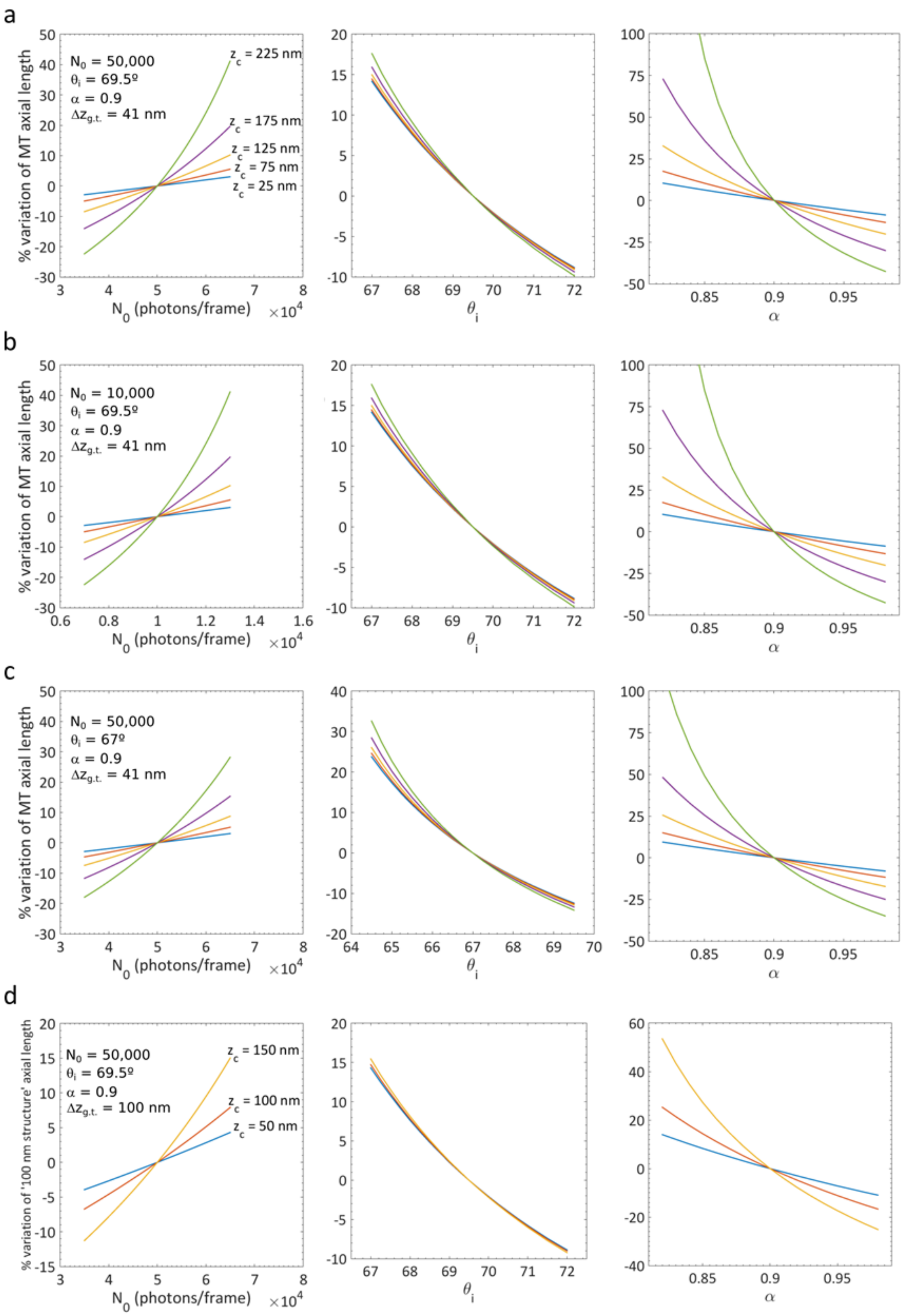

Supplementary Figure 5. Example percentual axial distortions introduced when using incorrect calibration parameters. (**a-c**) Percentual axial distortion of the SIMPLER image of a microtubule (41 nm diameter), centred at different axial positions, when incorrect values of N_0 (left), α (centre) and θ_i (right) are used. (**d**) Analogous calculations for a structure with 100 nm axial length. The values for each of the parameters are: (**a**) and (**d**) N_0 = 50,000 photons/frame; α = 0.9 and θ_i = 69.5°; (**b**) N_0 = 10,000 photons/frame; α = 0.9 and θ_i = 69.5°; (**c**) N_0 = 50,000 photons/frame; α = 0.9 and θ_i = 67°.

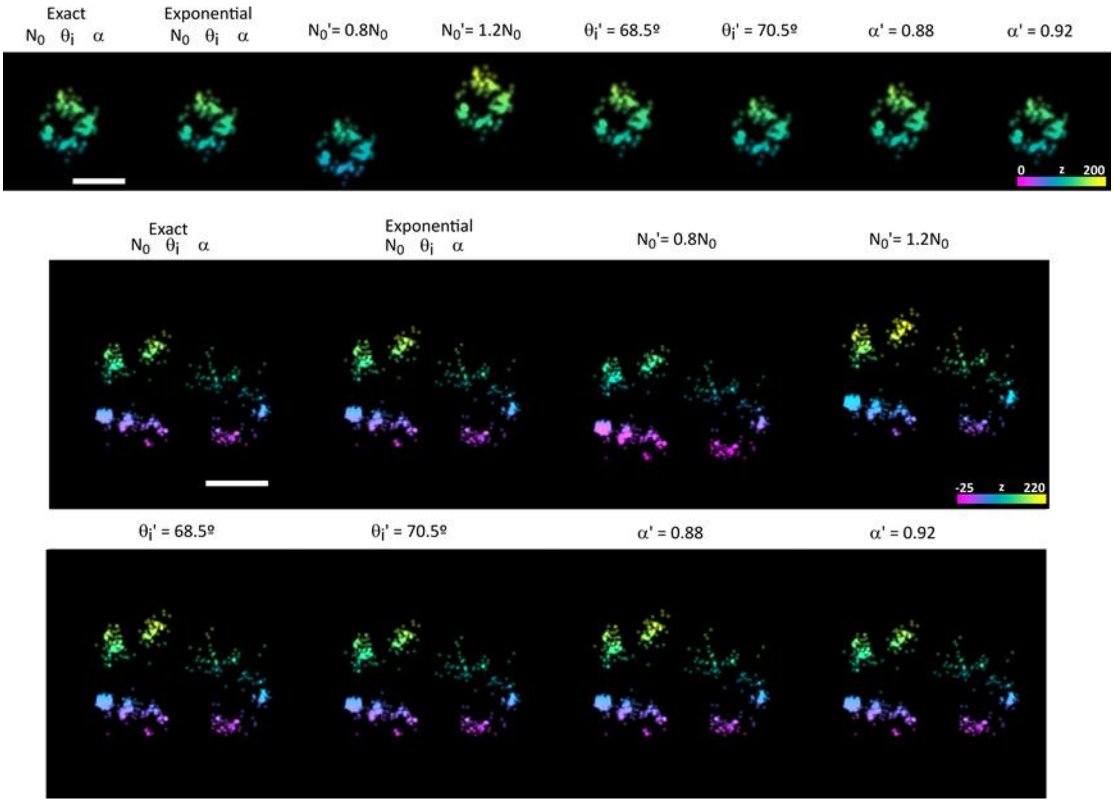

Supplementary Figure 6. Examples of SIMPLER reconstructions using different computation methods and varying α, θ_i, and N_0. Side views (*i.e. z-y* projections) of a spectrin ring (bottom) and a microtubule (top) obtained with different z -computation approaches. In the first images (left), z was computed numerically using the exact solution and $\alpha = 0.90$, $N_0 = 50{,}000$, and $\theta_\mathrm{i} = 69.5º$ as calibration input parameters. Right next to them, the results obtained with the exponential approach and the same input parameters are shown. Next, 6 different computations of the same data are shown for each structure, varying only one calibration input parameter per image ($N_0{}' = \{0.8, 1.2\}\, N_0$ for the first two examples; $\theta_\mathrm{i}{}' = \{68.5º, 70.5º\}$ for the third and fourth examples; and $\alpha' = \{0.88, 0.92\}$ for the last two cases). No significant axial distortions are observed over this range of parameters. The same slight effects were reproduced when we varied these parameters analysing the other two spectrin rings from Figure 2c and the other three microtubules from Figure 3a. Scale bars represent 50 nm (microtubules) and 100 nm (spectrin rings).

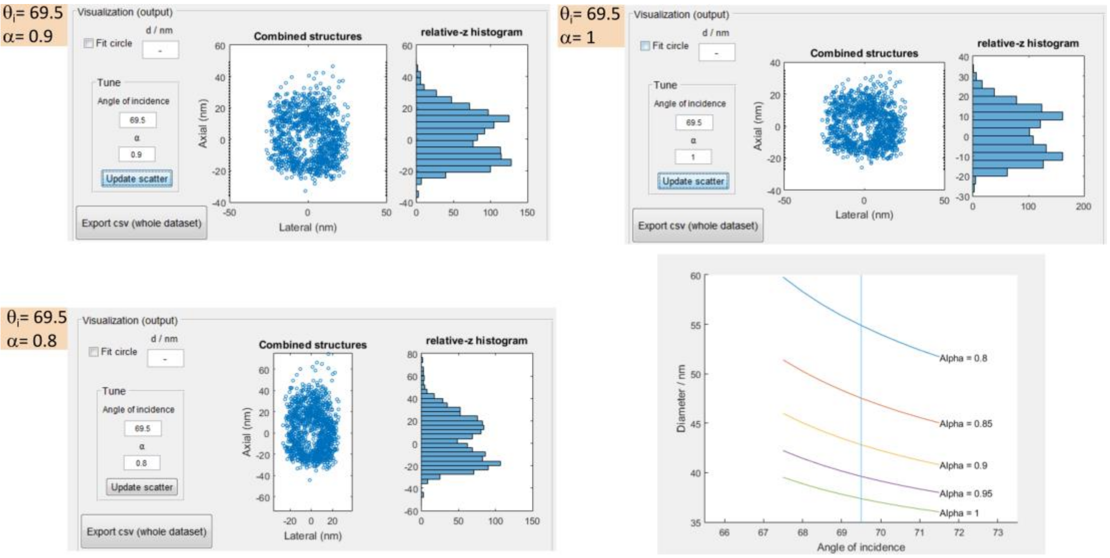

Supplementary Figure 7. Screenshot of the Supplementary Software for *angle and alpha tuning* operation output. This operation allows to visualize the combined images of well-defined structures varying either α or θ_i . In the shown examples, 8 cross-sections of microtubules centred at different axial depths are used (this example data is included within the software folder). It can be seen that, while the parameters $(\alpha, \theta_{\mathrm{i}})$ = (69.5º, 0.9) output a scatter plot with a circular aspect (as expected for a combination of microtubule's cross-sections), for $(\alpha, \theta_{\mathrm{i}})$ = (69.5º, 0.8) the combined image is axially elongated and for $(\alpha, \theta_{\mathrm{i}})$ = (69.5º, 1) it looks flattened. The software can perform a circular fit of the plotted data, and it is also possible to set a range of $(\alpha, \theta_{\mathrm{i}})$ values to automatically get a diameter from the circular fit for each condition and obtain a plot like the one shown on the figure (bottom right). In the latter, it can be seen that, for $\theta_{\mathrm{i}} = 69.5º$, a diameter in the 40-45 nm range is obtained for α between 0.9 and 0.95. Additionally, the other calibration input parameters (mainly N_0) can be varied before running this operation. Performing this analysis allows to find the best estimate for a parameter that have been determined or estimated with low accuracy. For further details, see the Software Documentation.

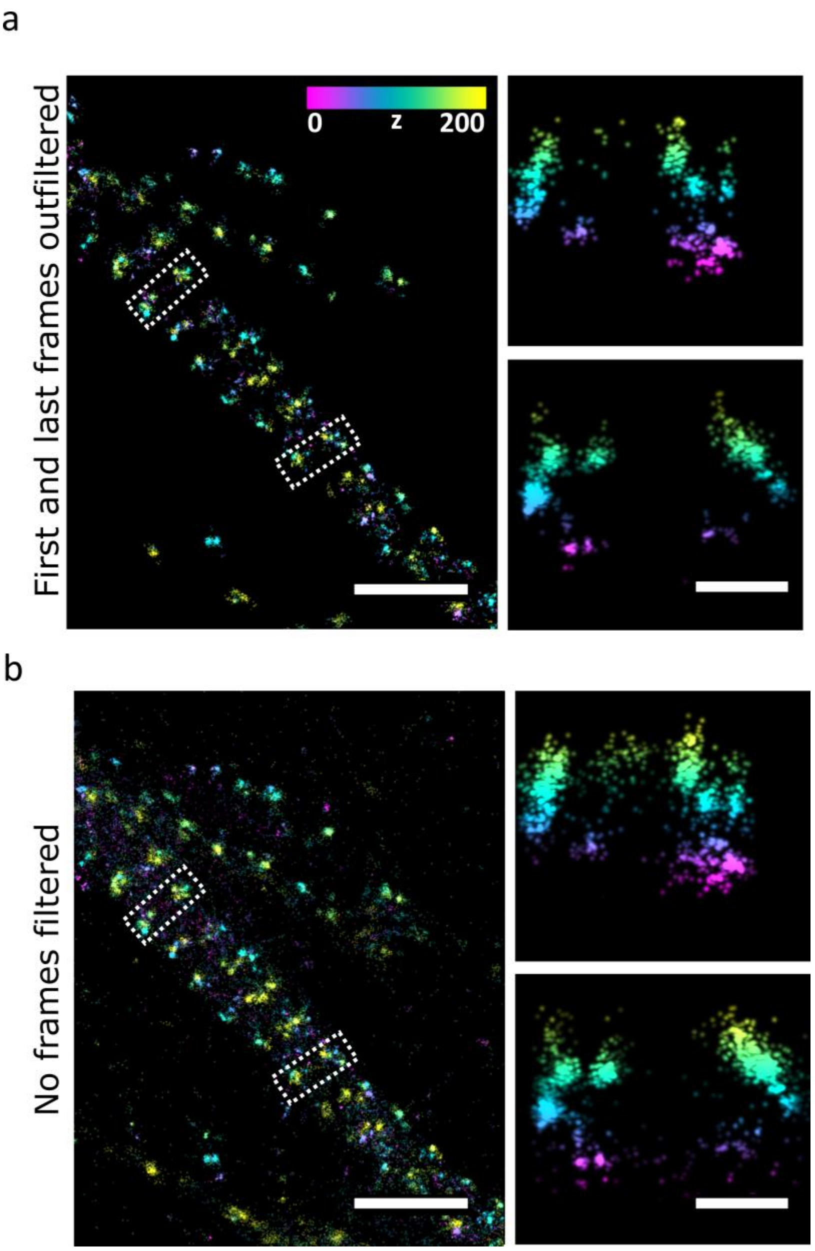

Supplementary Figure 8. Influence of the first and last frame filtering step on image quality for SIMPLER combined with DNA-PAINT. Overview image of β2-spectrin rings in neurons and magnified side-view reconstructions, *i.e.* *z*–*y* projections, of the boxed regions in the *x*–*y* view where the rendering was done with (**a**) and without (**b**) the frame filtering step of the localizations (described in Methods and Supplementary Fig. 1). In the *x*–*y* view, the filter's action resembles the one of a density filter, improving contrast by suppressing isolated or unspecific events. In the *z*–*y* projections, we see that the filter suppresses localizations that are wrongly assigned with higher z coordinate due to the incorrectly determined lower photon count. This effect is well reproduced in all the data we have analysed, including tubulin visualization in different biological samples (COS7-cells and hippocampal neurons), and the nuclear pore complex imaging in HeLa Kyoto cells. Scale bars represent 500 nm (top view) and 100 nm (side view). Number of localizations kept after the frame filtering step: 429 (top ring) and 652 (bottom ring). Number of localizations before performing the frame filtering step: 1186 (top) and 1573 (bottom).

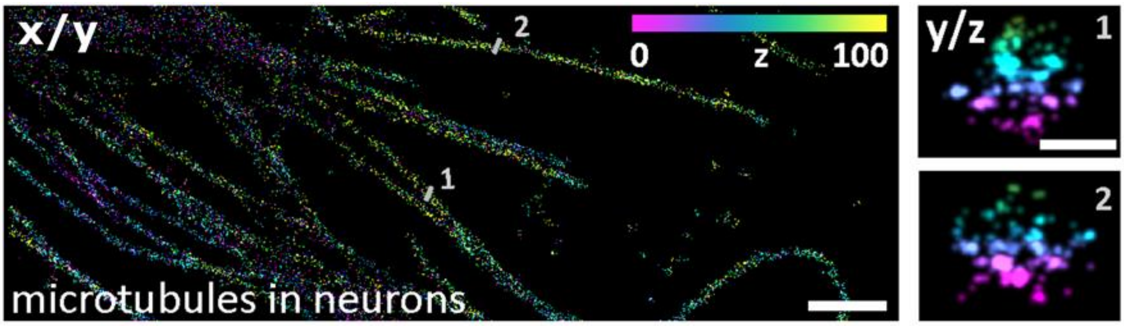

Supplementary Figure 9. Microtubules from hippocampal neurons immunolabeled for DNA-PAINT super-resolved in 3D using SIMPLER. Left: top view. Right: magnified side-views along the numbered line in the top view. We repeated this experiment two times with similar results. Scale bars represent 1 µm (top view) and 50 nm (side view). Number of localizations kept after frame filtering step: (1) 273 (out of 395) and (2) 168 (out of 472).

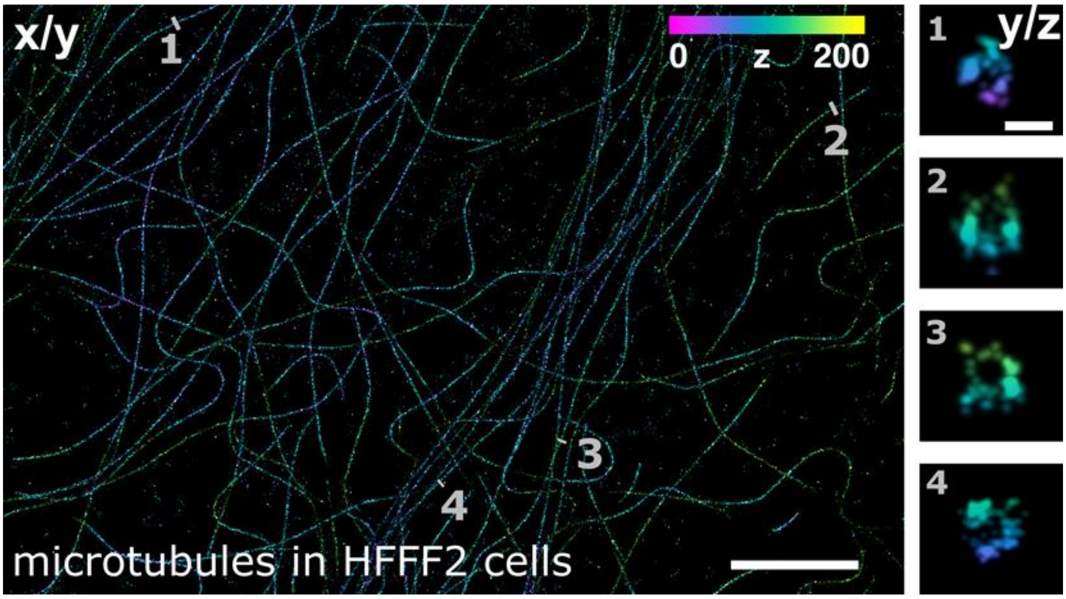

Supplementary Figure 10. Microtubules from Human Fetal Foreskin Fibroblasts cells, immunolabeled for DNA-PAINT super-resolved in 3D using SIMPLER using a commercial setup (Nikon STORM 5.0). Left: top view. Right: magnified side-views along the numbered line in the top view. We repeated this experiment three times with similar results. Scale bars represent 4 µm (top view) and 50 nm (side view). Number of localizations kept after frame filtering step: (1) 97 (out of 211), (2) 275 (out of 546), (3) 83 (out of 360) and (4) 126 (out of 302).

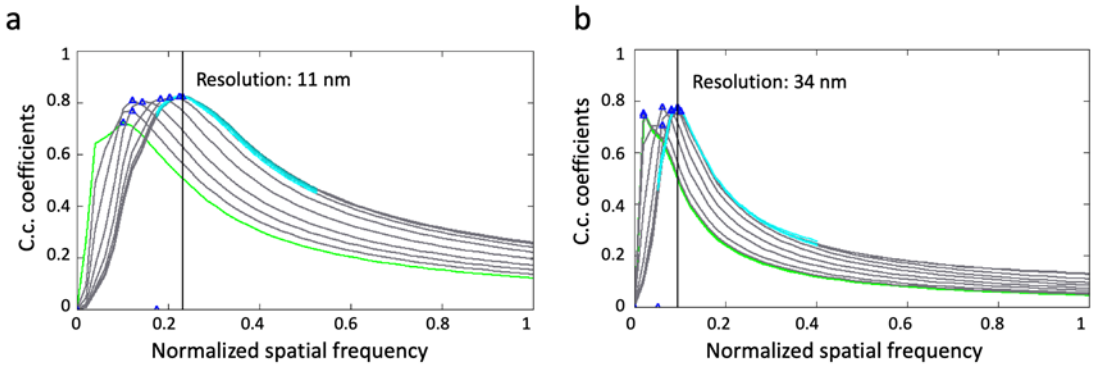

Supplementary Figure 11. Image decorrelation analysis. Decorrelation functions computed for the image-based resolution estimation for (**a**) DNA-PAINT images presented in Figure 3a and (**b**) dSTORM images presented in Figure 4. Green, decorrelation function without any high-pass filtering; grey, decorrelation functions with high-pass filtering; cyan lines, decorrelation functions with refined mask radius and high-pass filtering range; blue triangles, local maxima. Vertical line, cut-off frequency. C.c., cross-correlation.

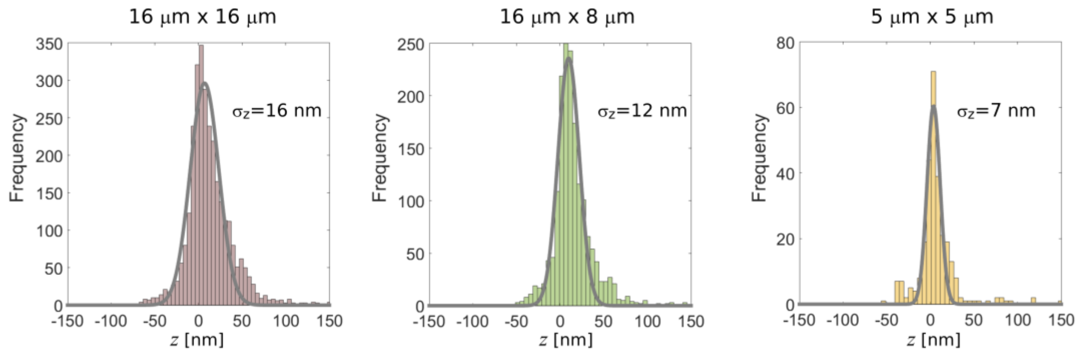

Supplementary Figure 12. Field-dependent z-uncertainty. Distribution of axial positions obtained with SIMPLER – DNA-PAINT for a sample of DNA-Fab fragments adsorbed to the coverslip (*z*-position of the fluorophores range from 0 to ~ 5 nm). From left to right: the uncertainty in *z* becomes smaller when the area of the region analyzed decreases.

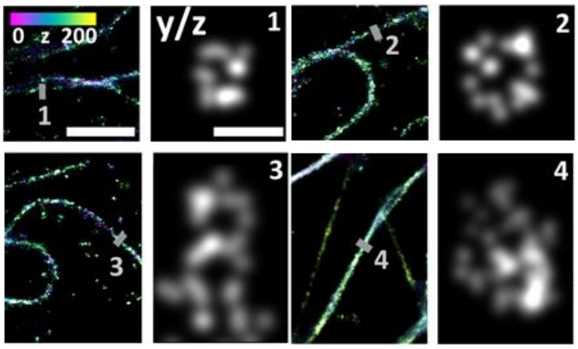

Supplementary Figure 13. Microtubules immunolabeled for dSTORM super-resolved in 3D using SIMPLER. (**a**) COS-7 cells (1 to 3) and hippocampal neurons (4). Color-coded: top view. Grayscale: magnified side-views along the numbered line in the top view. Scale bars represent 1 μm (top view) and 50 nm (side view). A N_0 value of 6,000 photons was determined. We repeated this experiment three times with similar results. Number of localizations kept after frame filtering step: (1) 20 (out of 43), (2) 19 (out of 201), (3) 36 (out of 179) and (4) 74 (out of 397).

Supplementary Tables

Parameter	Description	Main Effect when using an incorrect value
N_0	Emitted photons per frame by a fluorophore at z = 0	Axial offset. Axial distortions far from the surface (z > 150 nm); flattening if N_0 is underestimated, or elongations if N_0 is overestimated.
θ_i	Angle of light incidence	Axial distortions; elongations if θ_i is underestimated, or compressions if θ_i is overestimated.
$1 - \alpha$	Scattering contribution factor	Axial distortions far from the surface (z > 150 nm); flattening if α is overestimated, or elongations if α is under-estimated.

Supplementary Table 1. Effect of calibration parameters for axial determination via SIMPLER. Quantitative examples shown in Supplementary Figures 1, 5 and 6.

z [nm]		λ = 500 nm				λ = 530			
	NA	**1.4**	**1.42**	**1.45**	**1.49**	**1.4**	**1.42**	**1.45**	**1.49**
5		0.593	0.628	0.680	0.727	0.593	0.628	0.681	0.728
50		0.531	0.550	0.577	0.600	0.534	0.554	0.583	0.606
100		0.486	0.497	0.510	0.519	0.491	0.502	0.516	0.527
150		0.454	0.460	0.466	0.470	0.459	0.465	0.473	0.477
200		0.432	0.435	0.438	0.440	0.437	0.440	0.444	0.446
250		0.417	0.418	0.420	0.421	0.421	0.423	0.425	0.426
300		0.409	0.410	0.410	0.411	0.412	0.413	0.414	0.415
350		0.403	0.403	0.403	0.403	0.403	0.403	0.404	0.404
400		0.394	0.394	0.394	0.395	0.396	0.397	0.397	0.397
450		0.391	0.391	0.391	0.391	0.393	0.394	0.394	0.394
500		0.389	0.389	0.389	0.389	0.392	0.392	0.392	0.392

z [nm]		λ = 560				λ = 590			
	NA	**1.4**	**1.42**	**1.45**	**1.49**	**1.4**	**1.42**	**1.45**	**1.49**
5		0.593	0.629	0.682	0.729	0.594	0.630	0.683	0.730
50		0.537	0.558	0.588	0.612	0.540	0.562	0.593	0.618
100		0.495	0.507	0.523	0.535	0.499	0.512	0.529	0.542
150		0.464	0.471	0.479	0.485	0.469	0.476	0.485	0.492
200		0.441	0.445	0.450	0.452	0.446	0.450	0.455	0.458
250		0.425	0.427	0.429	0.431	0.429	0.431	0.434	0.436
300		0.416	0.417	0.418	0.419	0.419	0.421	0.422	0.423
350		0.406	0.407	0.407	0.407	0.410	0.410	0.411	0.412
400		0.400	0.400	0.400	0.401	0.402	0.403	0.403	0.403
450		0.396	0.396	0.396	0.396	0.398	0.398	0.398	0.399
500		0.391	0.392	0.392	0.392	0.394	0.394	0.394	0.394

z [nm]		λ = 620				λ = 670			
	NA	**1.4**	**1.42**	**1.45**	**1.49**	**1.4**	**1.42**	**1.45**	**1.49**
5		0.594	0.630	0.684	0.731	0.594	0.630	0.684	0.732
50		0.543	0.565	0.597	0.624	0.546	0.570	0.603	0.631
100		0.503	0.517	0.535	0.549	0.509	0.524	0.543	0.559
150		0.473	0.481	0.491	0.498	0.479	0.488	0.500	0.508
200		0.450	0.455	0.460	0.464	0.456	0.462	0.469	0.473
250		0.433	0.436	0.439	0.441	0.439	0.442	0.446	0.449
300		0.423	0.425	0.426	0.427	0.428	0.431	0.433	0.434
350		0.413	0.414	0.415	0.415	0.418	0.419	0.420	0.421
400		0.405	0.406	0.407	0.407	0.409	0.410	0.411	0.412
450		0.401	0.401	0.401	0.402	0.406	0.406	0.407	0.407
500		0.396	0.396	0.396	0.397	0.400	0.400	0.400	0.401

		λ = 700				λ = 720			
**z [nm]**	**NA**	**1.4**	**1.42**	**1.45**	**1.49**	**1.4**	**1.42**	**1.45**	**1.49**
5		0.598	0.634	0.688	0.735	0.593	0.630	0.684	0.732
50		0.546	0.570	0.604	0.632	0.549	0.574	0.609	0.638
100		0.509	0.524	0.544	0.560	0.514	0.530	0.552	0.569
150		0.479	0.488	0.500	0.509	0.485	0.495	0.509	0.518
200		0.456	0.462	0.469	0.474	0.463	0.469	0.478	0.483
250		0.438	0.442	0.446	0.449	0.445	0.449	0.454	0.458
300		0.428	0.430	0.433	0.434	0.434	0.437	0.440	0.442
350		0.417	0.418	0.420	0.421	0.423	0.425	0.427	0.428
400		0.409	0.410	0.411	0.411	0.414	0.416	0.417	0.417
450		0.405	0.405	0.406	0.406	0.410	0.411	0.411	0.412
500		0.402	0.402	0.403	0.403	0.404	0.404	0.405	0.405

Supplementary Table 2. Axial dependence of the collected fluorescence signal. Values of CF_{avg} as a function of z for various values of maximum emission wavelength (λ) and numerical aperture (NA), which together with the axial dependence of the illumination field allow users to extract the decay (d_F) and the background constant (α_F). Alternatively, users can directly obtain d_F and α_F by simply input of their experimental parameters in the supplemented MATLAB GUI (Supplementary Software 1).

Name	Sequence
Img1 (Imager strand)	ATTO655-5’-AGTTACATAC-3’
Img2 (Imager strand)	ATTO655-5’-AGAAGTAATG-3’
Dock1 (Docking strand)	5’-TATGTAACTTT-3’-Thiol
Dock2 (Docking strand)	5’-ATTACTTCTTT-3’-Thiol

Supplementary Table 3. DNA docking and imager strands sequences. All the oligos used were purchased from biomers.net GmbH.

Supplementary References

1. Schnitzbauer, J., Strauss, M. T., Schlichthaerle, T., Schueder, F. & Jungmann, R. *Nat. Protoc.* **12**, 1198–1228 (2017).
2. Ovesný, M., Křížek, P., Borkovec, J., Švindrych, Z. & Hagen, G. M. *Bioinformatics* **30**, 2389–2390 (2014).
