# Supplementary material for: Three-dimensional total-internal reflection fluorescence nanoscopy with nanometric axial resolution by photometric localization of single molecules
Source: Nat Commun. 2021 Jan 22;12:517. doi: 10.1038/s41467-020-20863-0 (PMC7822951; doi:10.1038/s41467-020-20863-0)
Supplement: Supplementary file 3 — Supplementary Software [file 41467_2020_20863_MOESM3_ESM.zip › SIMPLER Supplementary Software/Supplementary Software documentation.pdf]

Supplementary Software Documentation

SIMPLER

(Supercritical Illumination Microscopy Photometric z-Localization with Enhanced Resolution)

Summary 1

1. Input parameters 2
2. Filename and file format 4
3. Operations 4
   - i) Small ROI (r,z) (r = projection axis) 5
   - ii) Small ROI (x,y,z 10
   - iii) Large ROI 11
   - iv) N_0 calibration 11
   - v) Incidence angle & alpha adjustment (w/known structures) 12
   - vi) Calculate excitation profile from background 16
4. Visualization: Rendering 16
5. References 17

Summary

run_SIMPLER is a graphical user interface that runs in Matlab (version 2015a or later with Curve Fitting Tool installed). This app allows users of SIMPLER to perform all necessary operations to decode the axial positions of single molecules directly from 2D-SMLM-TIRF data. The software also includes modules to perform the following operations:

- Determination of N_0 from 2D-SMLM-TIRF data of emitters bound or adsorbed to the coverslip

- Calculation of the excitation intensity profile from 2D-SMLM-TIRF data of molecules spread all over the field of view; the list must include the background or offset information for each emitter.

- Adjustment of the calibration parameters θ_i, α, and N_0 using the SIMPLER 3D reconstructions of standard structures of well-defined, known geometry as feedback.

In this documentation, we include a detailed explanation of how to load files, set the different parameters, calculate *z*-coordinates of single molecules, and run the different operations available.

1. Input parameters/options

To run most of the available operations, the user will need to introduce the following parameters (Table SSD1).

Microscope	
i)	α **(alpha)**: It represents the fraction of the evanescent component of the excitation field at z = 0. Usually, it ranges from 0.85 to 0.92. We recommend setting it at 0.9 and, if necessary, perform an adjustment using reference structures with well-known geometry (*Operations* section 3.iv).
ii)	**NA**: Numerical aperture of the microscope's objective.
iii)	**Camera pixel size** (nm): This is needed to convert the sub-pixel position information from Picasso[1] files into *nm*, and to accurately perform the normalized Gaussian rendering.
Refractive index	
iv)	$\boldsymbol{n_i}$ (incident medium): Refractive index of the immersion oil.
v)	$\boldsymbol{n_s}$ (incident sample): Refractive index of the sample.
Fluorophore	
vi)	$\mathbf{N_0}$ (photons/frame): Number of photons emitted from a fluorophore located at z = 0 (interface) during the time-lapse of a single frame. Must be measured (see *N_0 calibration* from the *Operations* section).
vii)	**Emission wavelength** (nm): Maximum wavelength of the detected emission.
Excitation	
viii)	**Angle of incidence** (θ_i): Angle of incidence used in TIRF measurements (must be greater than θ_c). It must be measured or known from the instrument internal calibration (as is the case in a commercial TIRF setup). It can also be adjusted using reference structures as feedback (Operations section 3.iv)
ix)	**Excitation wavelength** (nm): Wavelength of the excitation laser.
Analysis	
x)	**Frame filtering; maximum (*x,y*) distance to link consecutive localizations (nm)**: In order to rule out localizations that represent emission events lasting less than the frame duration, the first and last frame of each single-molecule (SM) trace need to be discarded. This parameter sets the tolerance radius to consider two consecutive localizations belonging to the same molecule. We recommend a value between 15 and 20 nm.
xi)	**Correction due to non-flat illumination**: If checked, the software will perform an intensity correction to compensate local differences in the excitation intensity at each lateral (x, y) position due to non-uniform illumination. This option requires a file representing the excitation intensity profile (.csv file), whose size in RxC (rows x columns) coincides with the size of the field of view in camera pixels. In the .csv file, each cell will represent a specific camera pixel and its value will reflect the relative excitation intensity for that region. This information can be obtained, at least, in two ways. On one hand, we include an operation in which this profile can be obtained from a list of SM localizations spread all over the field of view, that must include the background information obtained from the MLE analysis (for a more detailed description, see *Calculate excitation profile from background* in the *Operations* section). Alternatively, the user can get this profile with an image of a solution of a fluorescent dye measured in the same conditions as the image that will be analysed by SIMPLER. The correction factor applied to each localization is calculated as "*max(excitation_profile)/excitation_profile(i,j)*", where *(i,j)* represents the camera pixel that contains the centre of the localization. By doing this, the photon count of every localization is

	converted to the value that it would have if the illumination was flat and with an intensity equal to its maximum value.

Table SSD1. Description of the input parameters / options needed to perform SIMPLER with *run_SIMPELR* software.

Figure SSD1 shows a screenshot of the app, where all the described input parameters can be seen.

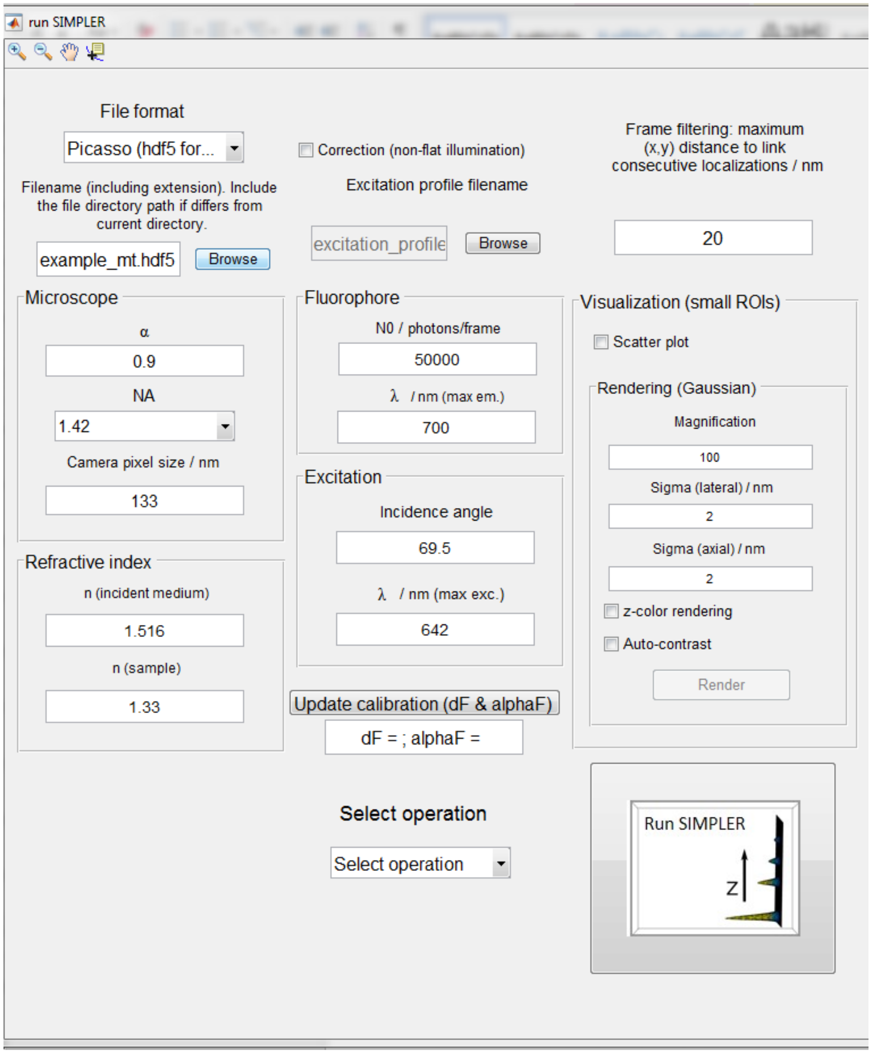

Figure SSD1. *run_SIMPLER* Matlab graphical user interface. The input parameters i) to xi) are shown in this screenshot.

Parameters i), ii), iv), v) and vii) to ix) determine dF and alphaF (α_F and d_F). The user can press the *Update calibration output* button to check the values of dF and alphaF that would be obtained with the current parameters.

Parameter v) **N_0** is needed to fully achieve the calibration equation (Eq. 2 from the SIMPLER manuscript) and decode z data from the 2D SMLM information.

2. Filename and file format

run_SIMPLER is capable of reading SMLM lists files from Picasso (.hdf5 format) and ThunderStorm[2] (.csv). If the user has used another software to perform MLE analysis, we also include a third option where a .csv custom-built file can be loaded. These three options are accessible from the pop-up menu that appears below *File Format* (Figure SSD1).

The minimum information needed from every SM to perform SIMPLER is a list of localizations including *x position*, *y position*, *frame number*, and *number of photons*. If the user wants to obtain the excitation profile from a SMLM list, the *background* (Picasso) or *offset* (Thunderstorm) information is also needed (see the description of parameter xi) in the *Parameters/options section*, and *Calculate excitation profile from background* in the *Operations* section).

If the user decides to use a .csv custom file, he/she will get the following message: "*You have chosen "Other" csv file format. Please make sure that the order of the columns in your list is as follows: 1) frame, 2) x (nm), 3) y (nm), 4) intensity (photons) and 5) offset or background (photons; only needed if running the "Calculate excitation profile from background" operation). Also, remove headers. Once verified, click Ok to continue.*" Thus, the user must ensure that the columns are organized this way.

It must be noticed that if the file is located in a different folder from the *run_SIMPLER* directory, the filename must include the full file directory path. If the user uses the *Browse* button, the full directory will be included by default.

Finally, if the *Incidence angle & alpha adjustment (w/ known structures)* operation is selected (see the *Operations* section for a more detailed description), the reference structures' file must be organized as follows: (i) each individual structure must have one column with the lateral position and another with the axial (*x/z, y/z* or *r/z*, where "*r*" is a combination of *x* and *y*); (ii) if the user has measured *N* individual structures (and obtained their axial positions with SIMPLER), then the .csv file will have *2xN* columns; (iii) odd columns (i.e., *j* column) will contain the lateral position, while even columns will be filled with the axial information (i.e., *j+1* column).

3. Operations

run_SIMPLER includes several operations (Figure SSD2). The first three options are for z-calculation in i) (*Small ROI (r,z), ii) Small ROI (x,y,z), and iii) Large ROI*. The fourth operation (N_0 *calibration*) delivers an N_0 value from a list of photon counts of SM located at z = 0. The fifth operation (*Incidence angle & alpha adjustment*) allows performing an adjustment of the calibration parameters based on the SIMPLER 3D reconstruction of known structures, and the last (sixth) operation (*Calculate excitation profile from background*) permits users to obtain a .csv excitation profile file from a list of localizations. In this section, we will explain each operation and show how to run them by analysing available example data that has been used in the SIMPLER manuscript.

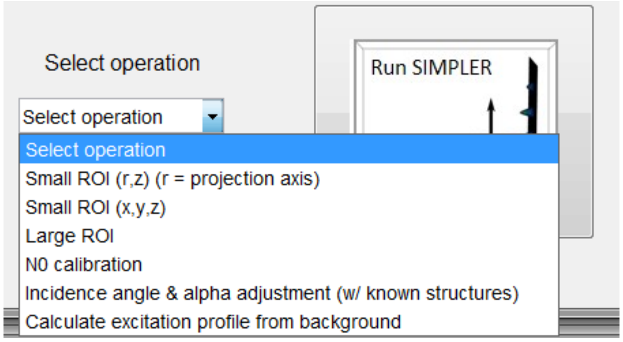

Figure SSD2. Operations pop-up menu.

i) Small ROI (r,z) (r = projection axis)

This operation was designed to analyse small regions, where the user is interested in visualizing a cross-section that does not coincide with the canonical *x*- or *y*-axis. In the manuscript, we used it to visualize the cross-section of microtubules and single spectrin rings. Hereafter, we will refer to the lateral direction of the cross-section as the "*r-axis*" (instead of *x*- or *y*-).

The software automatically defines this new axis by performing a linear regression of the *(x,y)* data, as schematically shown in Figure SSD3. The "r_N" position of each molecule (i.e. the projection of each point into the *r* axis) is obtained using trigonometry. The scheme from Figure SSD3 depicts this entire procedure. Next, we will show how this operation works with an example of microtubules and one of spectrin rings.

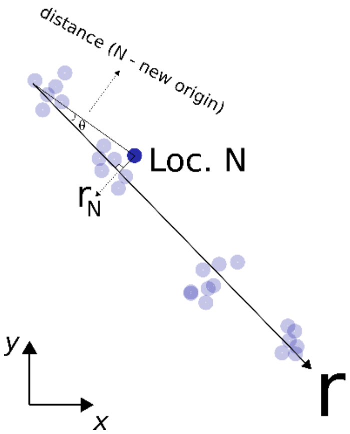

Figure SSD3. Scheme of a scatter plot of the *(x,y)* data from the ROI selected to be analysed with the "*Small ROI (r,z) (r: projection axis)*" operation.

Example 1: *microtubules (microtubule #1 from Figure 3 of the SIMPLER article)*

Figure SSD4 shows three different screenshots of the Picasso "Render" module, where the SMLM data corresponding to microtubules in COS7-cells from Figure 3 of the SIMPLER manuscript (+10M localizations) is depicted in red. From left to right, a progressive zoom-in is performed until selecting the small ROI to be analysed ("picked", in terms of Picasso software) (light blue/white). We provide this example data in file "example_mt.hdf5" in the *run_SIMPLER* folder. The blue rectangle shows the orientation of the axis that joins the selected localizations. As explained above, this orientation can be obtained from a linear fit of the *(x,y)* positions from the picked localizations. We have selected these localizations intentionally to define a direction approximately perpendicular to the orientation of one of the microtubules, and to obtain a suitable *r*-axis for microtubule cross-sections.

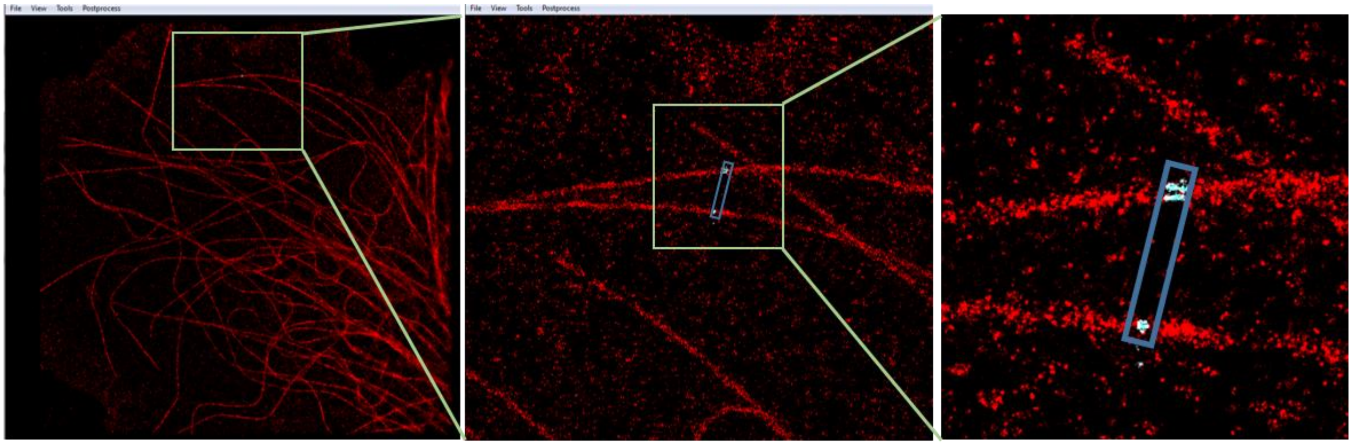

Figure SSD4. SMLM data from microtubules in COS7-cells rendered in Picasso "Render" module. The entire dataset is red-coloured, while the picked localizations (those chosen to be axially analysed) are colored in light blue/white. The second and third images correspond to zoom-in visualizations of the green rectangles from the first and second images, respectively.

In Figure SSD5, we show a screenshot of the *run_SIMPLER* app with the adequate parameters introduced for this example (additionally, an .xlsx file with these parameters is included in the *run_SIMPLER* folder). We have also checked the "*Correction due to non-flat illumination*" box, added the corresponding excitation profile filename (the file is also included in the program folder), and clicked on the "*Scatter plot*" visualization option.

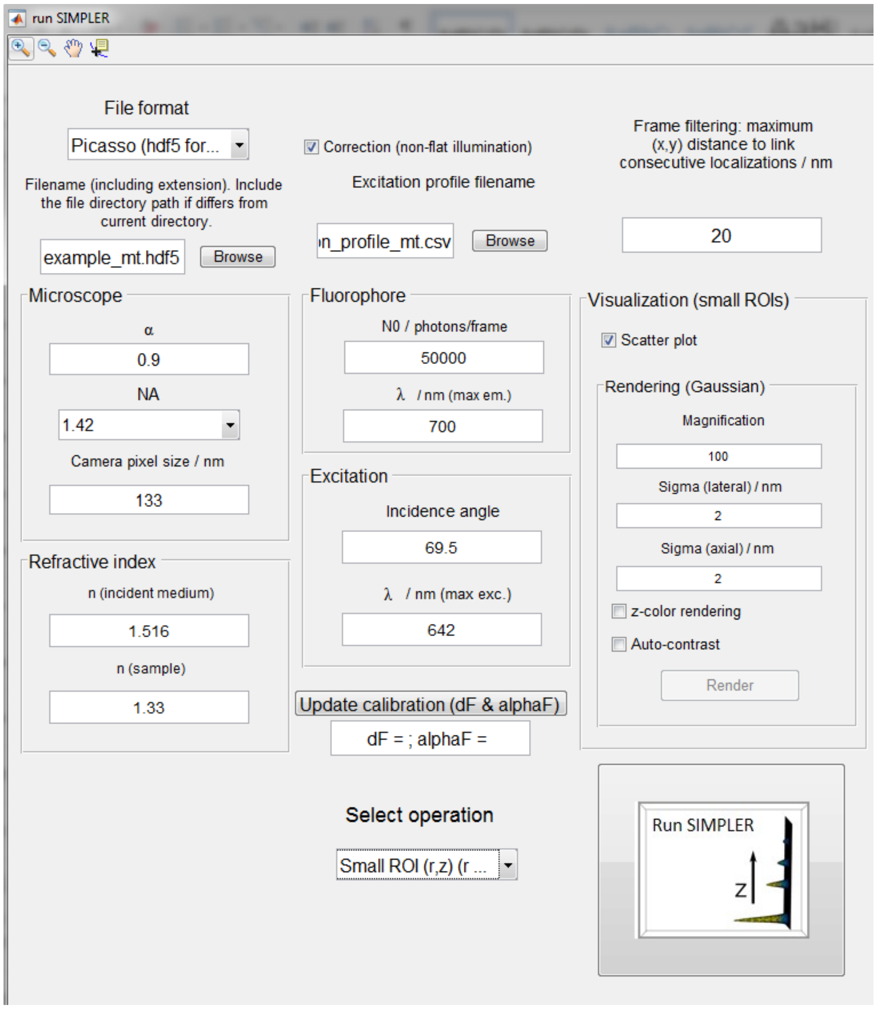

Figure SSD5. *run_SIMPLER* screenshot with correct parameters loaded for the microtubule's example data.

Once the parameters are set, the user can run the *z*-localization script by clicking on the *Run SIMPLER* button (bottom right). At this point, it should be clarified that, before performing the frame filtering step, SIMPLER removes duplicates that could have been artificially generated by the procedure of selection of data. These events are detected because they have exactly the same (*x, y, frame, photon count*) values. When the analysis has finished, a message box will inform the user the total number of localizations from the raw data, together with the number of valid localizations kept after the filtering step, as shown in Figure SSD6. Also, as we have checked the "Scatter plot" box before running the analysis, a *z vs. r* scatter plot will be presented in the visualization box.

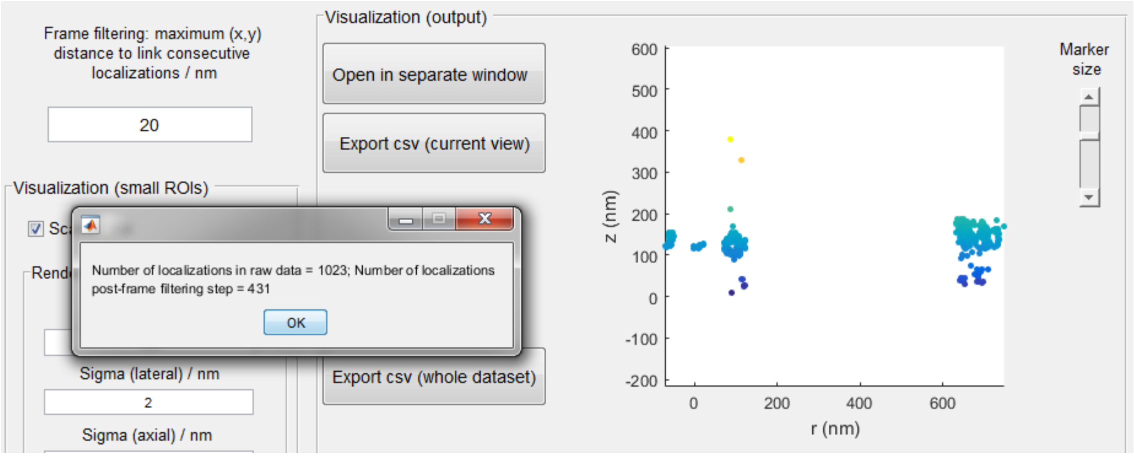

Figure SSD6. Message box informing the filtering step result (# original events, # valid (or kept) events). Behind this box, the scatter plot can be seen in the visualization box.

With the zoom tool that can be found on the top left of the app, the user can navigate the scatter plot, which is set to keep a data aspect ratio of 1:1 between the *z* and *r* axis (i.e. even if you perform a zoom in, both axis will still be set at the same scale so the scatter plot is not distorted). In Figure SSD7 we show the (*r,z*) position where the example microtubule #1 from Figure 3 of the SIMPLER manuscript is located. As it can be seen in the same figure, there is an option to open the scatter plot in a separate window, a button to export as .csv the current view, and also an option to export the whole dataset. When the user is performing this operation (*Small ROI, (r,z)*), two exported files will be generated: one with the (*x,y,z*) information, and another one with the (*r,z*) data. The format of the exported files includes the adequate headers to let the user import the data in ThunderStorm.

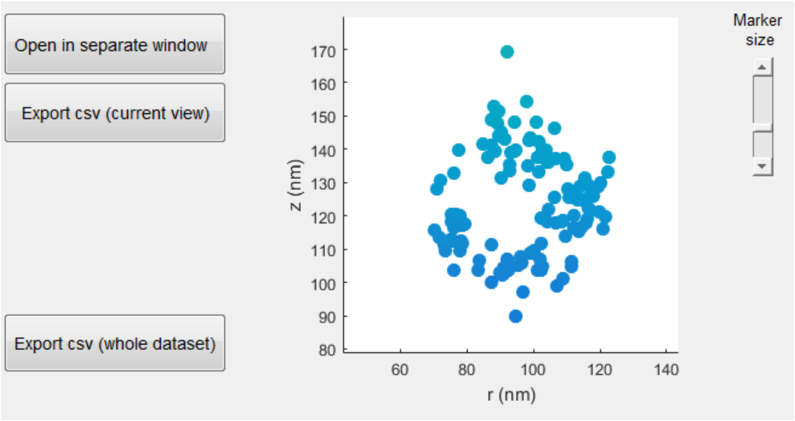

Figure SSD7. Message box informing the filtering step result (# original events, # valid (or kept) events). Behind this box, the scatter plot can be seen in the visualization box.

<u>Example 2</u>: spectrin ring (ring #3 from Figure 2d of the SIMPLER manuscript)

Figure SSD8 shows two different screenshots of the Picasso "Render" module, corresponding to *(x,y)* visualizations of the spectrin data (Figure 2b of the SIMPLER manuscript). Again, the small ROI selected to be

analysed is shown in light blue/white, while the whole spectrin data is plotted in red. The inclined blue rectangle from the second image coincides with the *r* axis that is obtained when performing a linear regression of the (*x,y*) positions of the selected data.

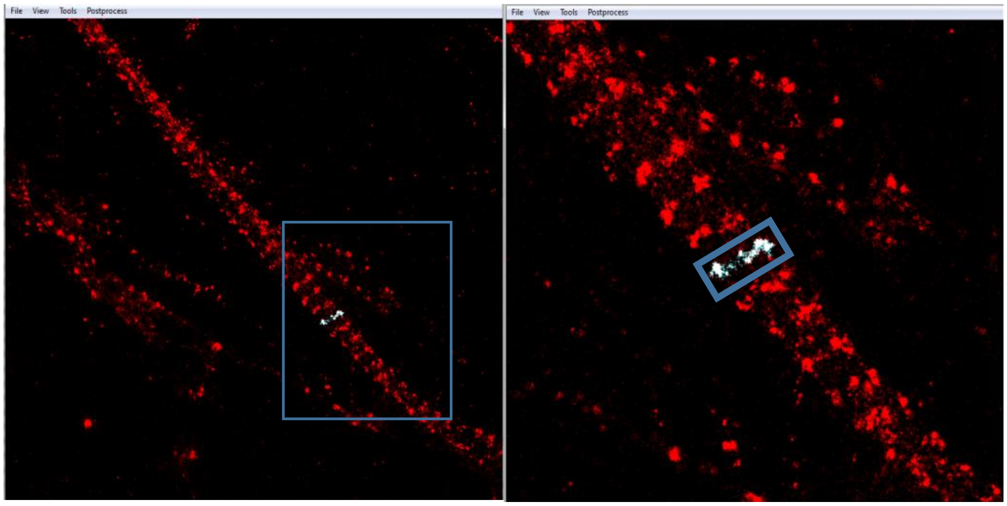

Figure SSD8. SMLM data from spectrin in hippocampal neurons, rendered in Picasso "Render" module. The entire dataset is red-coloured, while the picked localizations (those chosen to be axially analysed) are coloured in light blue/white.

In Figure SSD9 we show the results obtained for this example (data provided in files "example_spectrin.hdf5" and "excitation_profile_spectrin.csv").

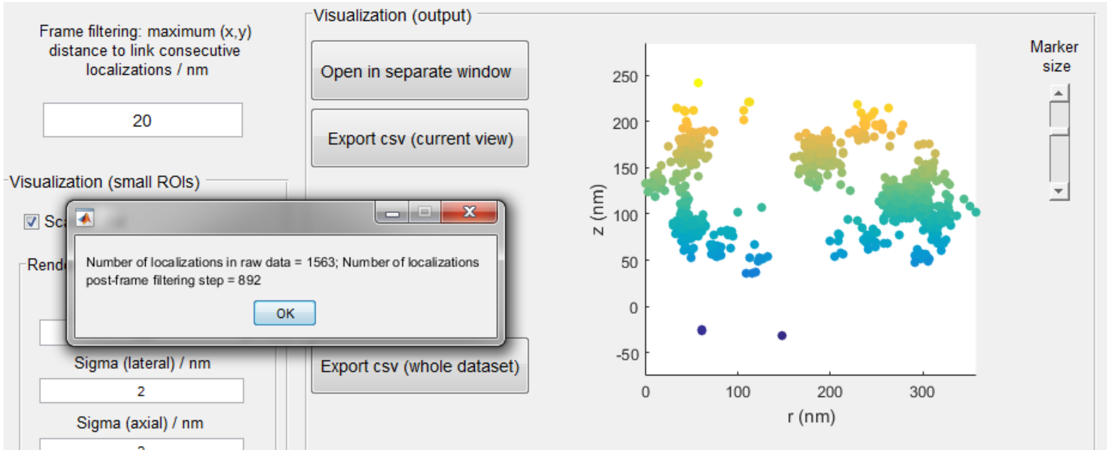

Figure SSD9. Message box informing the filtering step result (# original events, # valid (or kept) events). Behind this box, the scatter plot can be seen in the visualization box.

ii) Small ROI (*x,y,z*)

We have shown in the previous two examples how to use *run_SIMPLER* to both get *z* information and a projection axis which differs from the *x*- or *y*-axis, by choosing the "*Small ROI (r,z) (r: projection axis)*" operation. However, if the user is not interested in getting a cross-section and only wants to calculate the axial position of a list of localizations corresponding to a small ROI, we recommend the "*Small ROI (x,y,z)"* operation. Next, we show how the output of this operation looks like with an available example (one of the 4 nuclear pore complexes used for the averaged image from Figure 4b of the SIMPLER manuscript).

<u>Example 3</u>: Nuclear Pore Complex (one of the NPC used for the averaged image from Figure 4b of the SIMPLER manuscript)

This example was measured in a different microscope and with a different technique (dSTORM). The input parameters should be modified accordingly. In Figure SSD10 shows the correct parameters (left) and the visualization output (right). Additionally, you can find these parameters in the .xlsx provided in the software folder. As it can be seen, there are now two different scatter plots: one for the *(x,z)* side view and one for *(y,z)*.

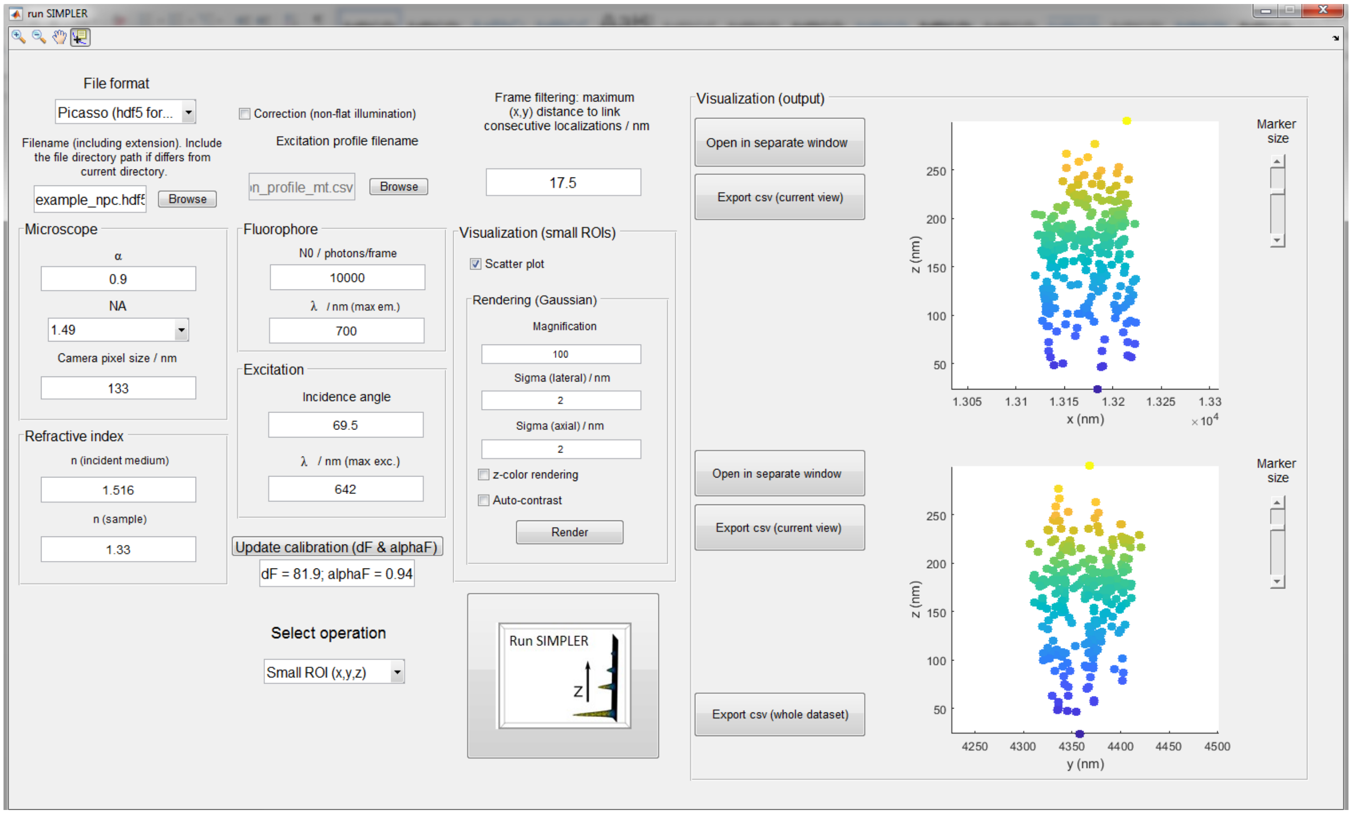

Figure SSD10. Correct input parameters to perform "*Small ROI (x,y,z)*" operation to NPC example data (left). Scatter plot of the side views (*x,z*) and (*y,z*) (right).

iii) Large ROI

This operation requires the same inputs as those described for small ROIs. The data gets automatically exported to .csv file. We recommend this operation when the goal is to analyse large fields of view. If the scatter plot box is checked, an *(x,y)* scatter plot will appear after the analysis is complete. A small rectangular (and rotatable) ROI can be selected from this scatter plot of the top view, in order to visualize an '*x-z*' or '*y-z*' side view in a lower panel (Figure SSD11).

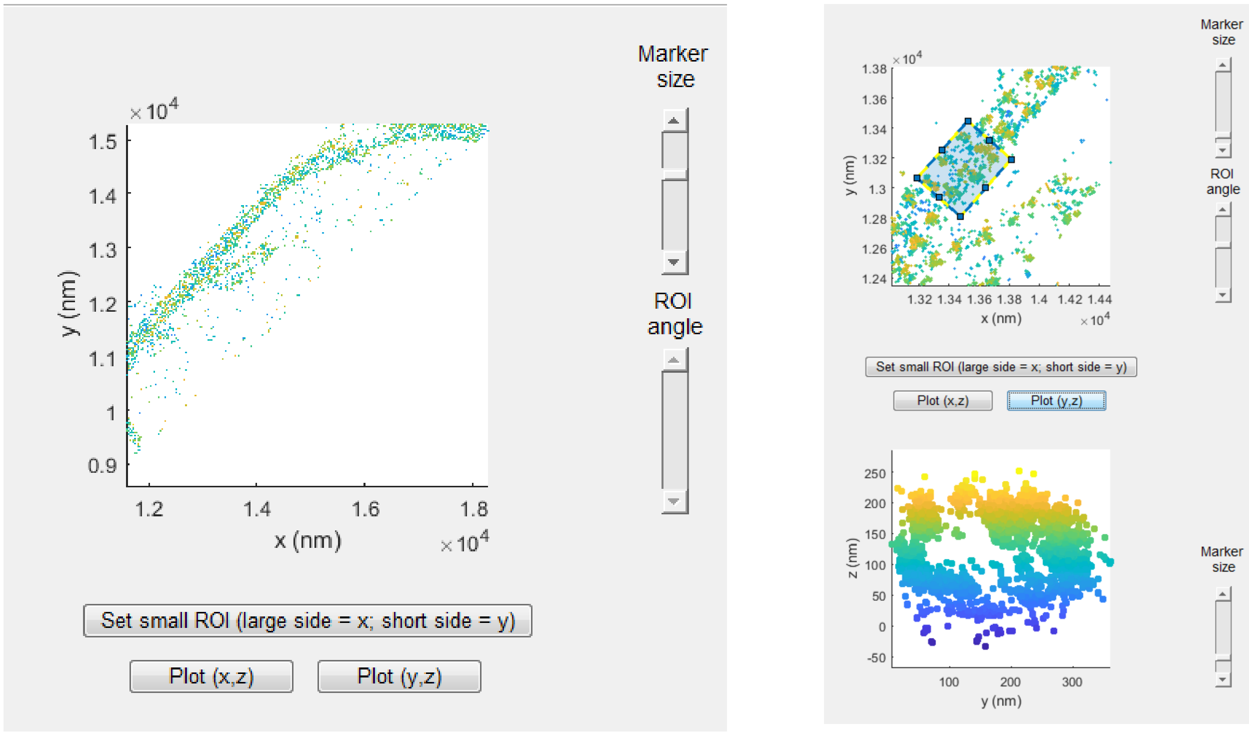

Figure SSD11. Visualization of *(x,y)* data from a "Large ROI" (whole dataset on the left and zoom in on top-right) and of *(y,z)* points from the selected ROI (bottom right).

iv) N_0 calibration

One key parameter of SIMPLER is N_0, i.e., the number of emitted photons per frame for a fluorophore located at the surface. We provide here an operation to directly obtain this value from a list of 2D-SMLM-TIRF data obtained from fluorophores at, or very close to, the surface (e.g. by imaging antibodies adsorbed to the coverslip, a flat DNA-origami or DNA-docking strands or fluorophores directly bound to the surface through a biotin-streptavidin strategy). When this operation is selected, the program does not perform a *z*-calculation. Instead, it outputs a histogram of the number of photons per frame together with the results of a 1-peak Gaussian fit. The main advantages of getting the N_0 value through this operation, and not just from looking at the raw data, is two-fold. On the one hand, we allow the user to perform the correction due to non-flat illumination, and on the other, we remove invalid localizations from the analysis with the frame-filtering step. In Figure SSD12 we show the results of running the N_0 calibration to the example data "example_Fab.hdf5" file, which corresponds to the Fab

fragments imaged to obtain the N_0 shown in Figure 2b from the SIMPLER manuscript (the excitation profile file is called “excitation_profile_fab.csv”).

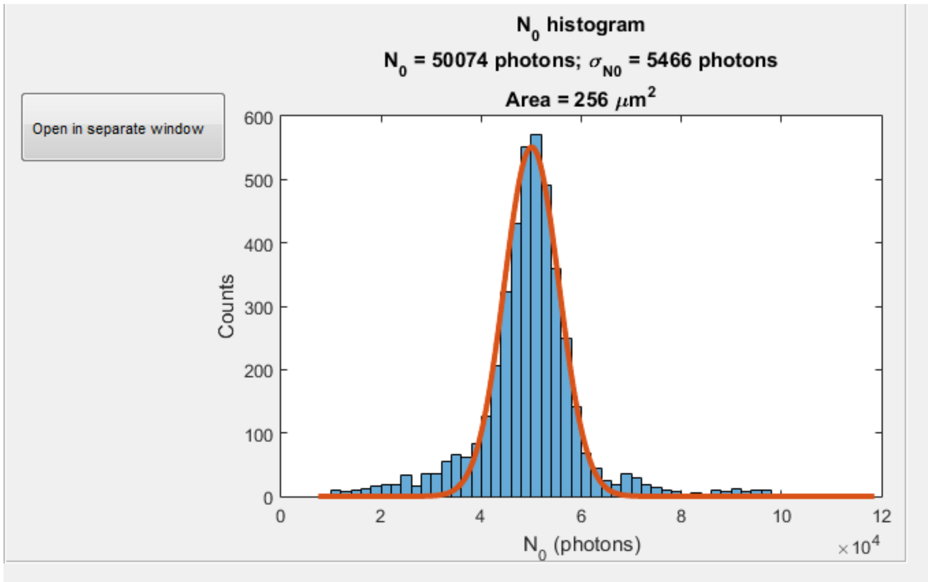

Figure SSD12. Results obtained for the “*N_0 calibration*” operation.

v) Incidence angle and alpha adjustment (w/known structures)

If desired, the user can perform an adjustment of the calibration parameters by evaluating the resulting 3D reconstruction of reference structures of known geometry. Particularly, the angle of incidence (θ_i), and alpha (α) can be optimized. Inaccurate values of θ_i can introduce axial distortions throughout the whole TIRF range. Contrary, incorrect values of α or N_0 can generate axial distortions only for structures located far from the surface. These behaviours are described in Supplementary Figures 1, 5, and 6 of the SIMPLER manuscript.

The Incidence angle and alpha adjustment operation was designed for using two types of reference structures: a structure of circular cross-section (such as the microtubules), or a structure with two well-defined z levels (such as the nuclear pore complex or a DNA origami). Below we describe how to use this operation and test it with the example data provided.

The user must load a .csv file containing localizations from *N* individual known structures. Ideally, the structures must represent different axial positions within the TIRF range. These localizations will represent side views, i.e. for each object the user must count with two columns with the (*x,z*), (*y,z*) or (*r,z*) information. In order to see how to organize the localizations from numerous objects into a single .csv file, please go to the *Filename and file format* section. In the *run_SIMPLER* folder we include an example with 8 microtubule cross-sections

named “example_mt_rz_tunning_8mts.csv”. After setting all the input parameters, the user can click on the *run_SIMPLER* button. The Visualization box will look like the screenshot shown in Figure SSD13.

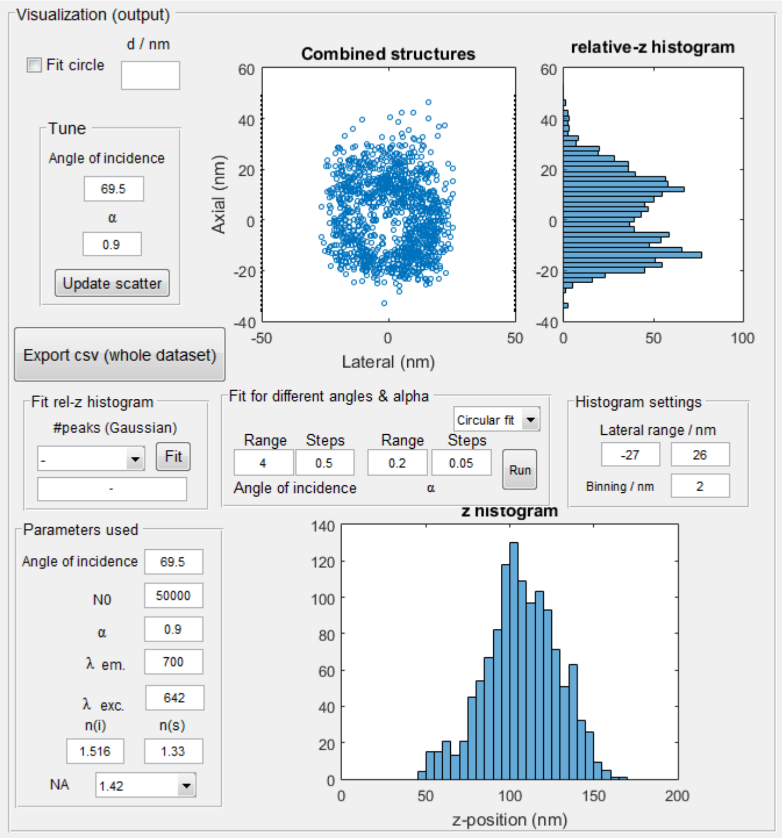

Figure SSD13. Screenshot of the “Parameters adjustment” output.

As shown on the bottom of Figure SSD13, a list of the used parameters is displayed. These text boxes are inactivated and, thus, cannot be modified. Next to the *Parameters used* panel, a histogram of all the *z*-positions is plotted. This distribution indicates the axial range from where the localizations were obtained. In this case, the spanned *z* range was ~ 50-160 nm.

On the top of Figure SSD13, a scatter plot of the combined structures is presented. In order to move every structure to the same origin, the software calculates within each structure a central (*lateral, axial*) position this way: for both dimensions, it finds the lateral (or axial) position corresponding to the 10- and the 90-percentile event, and obtains the “central” point by calculating the mean value between those 10/90 percentile localizations. Then, to find the relative coordinates, the central lateral value is subtracted to each lateral position of the structure, and the same is done for the axial direction with the central axial value. We found this operation more robust than finding a centroid or a centre of mass of each object, because some structures can be incompletely labelled and then these centre estimators may be less confident. Our approach is similar to take the mean value between the maximum and minimum values within the distribution, but by using the 10 and 90 percentile we avoid outlier artifacts and find a better centre estimate.

On the right of the scatter plot, a relative-z histogram is shown. The vertical axis of this histogram coincides with the axial axis of the scatter plot. By default, all the localizations are included in this analysis, but the user can

modify the lateral limits, as shown in Figure SSD14, where the “Lateral range” (bottom right) is set to be -7 / 3 nm (the vertical dotted lines in the scatter plot represent these limits).

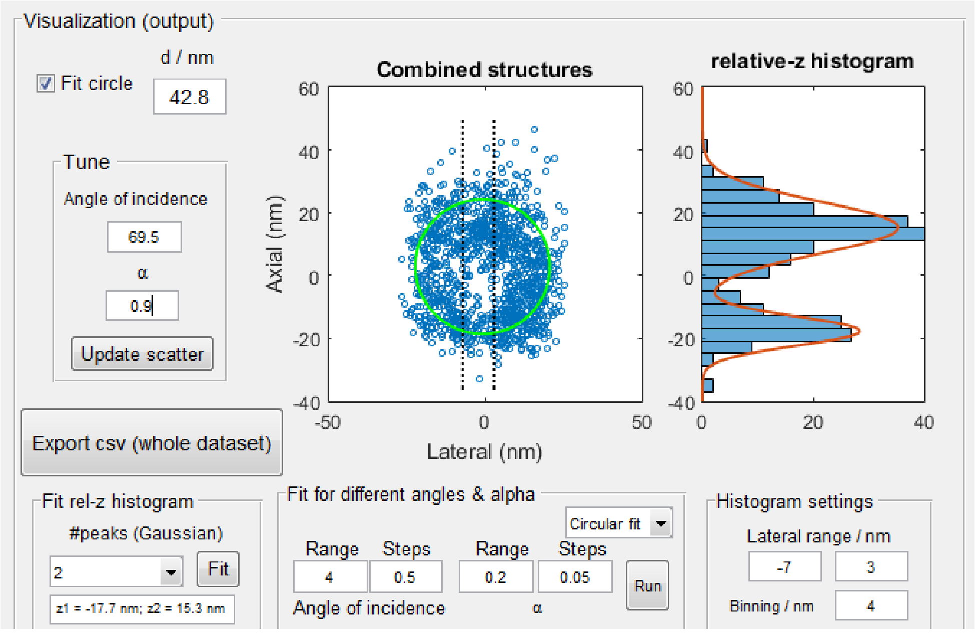

Figure SSD14. Screenshot of the scatter plot of combined structures (microtubules), with the “Fit circle” activated and the relative-z histogram fitted by a 2-peak Gaussian function.

We provide two types of operations to analyse the combined data: on one hand, a circular fit can be performed by checking the *Fit circle* box, as depicted in Figure SSD14 (in this case, a 42.8 nm diameter is found); alternatively, the relative-z histogram can be fitted by a 2-peak Gaussian function (bottom left, Figure SSD14). We find the *Fit circle* option more adequate for microtubules cross-sections, and the 2-peak Gaussian function more suitable for other kinds of structures with 2 well-defined levels (e.g. NPC, DNA-origamis). The *Tune* panel (Figure SSD14, left) lets the user manually modify both the incident angle and the α values, and by clicking on “Update scatter” the z-values are re-calculated and retrieved into the scatter plot.

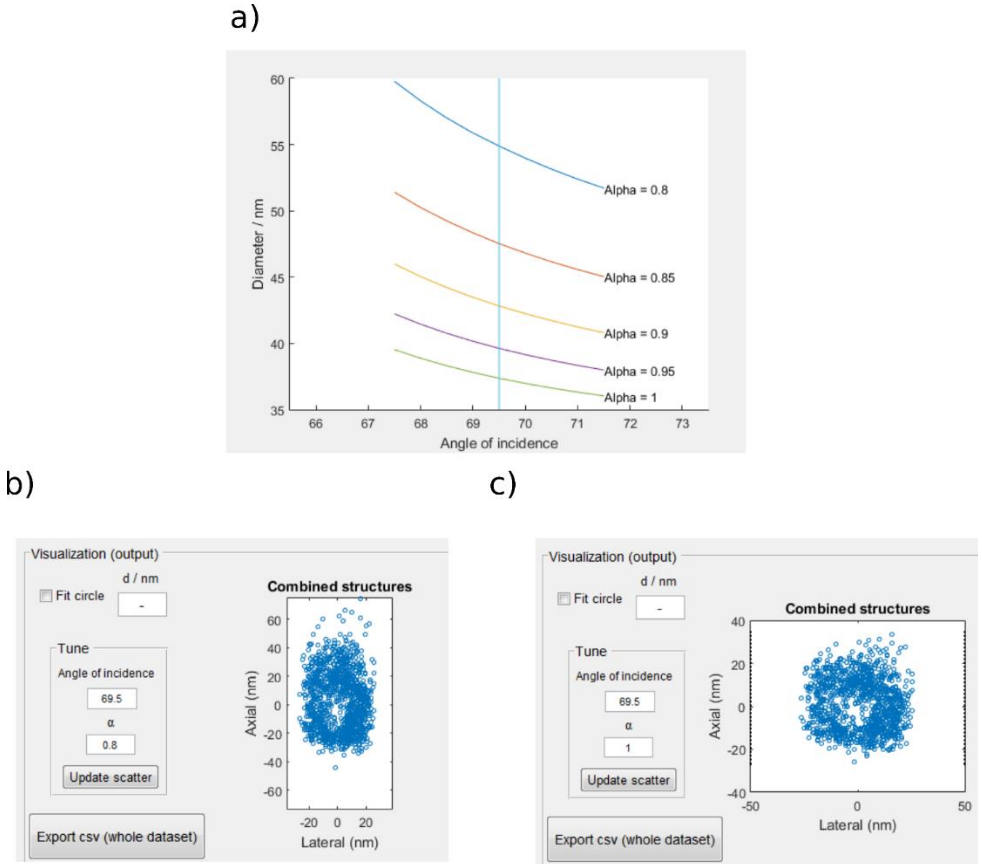

Figure SSD15. a) Output figure of the automatic parameters tuning operation. The diameters obtained after circular fits for different angles and alpha values are plotted. b) and c): Scatter plot of the combined microtubules' cross sections setting (θ_i, α) to (69.5, 0.8) and (69.5, 1), respectively.

The user can also explore different (θ_i, α) values by defining a certain range and steps for each parameter in the *Fit for different angles and alpha* panel (Figure SSD14, bottom centre). Furthermore, the user can choose to analyse each condition with a circular fit or with a 2-peaks Gaussian fit. After setting the parameters and clicking on *Run*, the software will scan all the conditions and output a figure containing a plot of either (z_2-z_1) (for 2-peak Gaussian fit) or radius (for circle fit) vs θ_i for different α. In Figure SSD15.a we show an example of this output. In this case, a diameter between 39.6 and 42.9 nm is obtained if θ_i is set to 69.5º (as measured) and α spans from 0.9 to 0.95. If we move to α = 0.8 (or 1), the diameter takes a 54.8 nm (37.3 nm) value, which is higher (lower) than what is expected for microtubules immunolabelled with primary antibodies and Fab fragments from secondary antibodies. Additionally, if we check the scatter plot for (θ_i, α) = (69.5, 0.8) or (69.5, 1), we confirm that the combined structures look either too elongated or too flattened, respectively (Figures SSD15 b and c). Naturally, the *Incidence angle and alpha adjustment (w/known structures)* operation can be modified to use other geometric parameters as feedback or perform other type of analysis. All operations where axial positions are calculated allow the user to export as .csv list the (*lateral, axial*) information achieved after performing SIMPLER.

vi) Calculate excitation profile from background

This operation allows users to obtain the information about the local excitation intensity at each lateral position due to uneven excitation intensity. This information is necessary to correct the photon counts of single molecules when the illumination used is non-flat (e.g. Gaussian beam), as described in the SIMPLER manuscript.

The software takes as input the local background of single molecules from a list of 2D SMLM localizations (*offset* in ThunderStorm, *background* in Picasso). The local background value is informed in photon counts per frame, both from Picasso or ThunderStorm. If the user performs the 2D analysis with another software, he/she must ensure that these values are actually informed in photon counts per frame. Running the *Calculate excitation profile from background* operation assigns to each camera pixel a value that is the average of the *offset* (or *background*) parameter obtained from every localization belonging to that pixel.

Ideally, the input list of localizations should cover the complete field of view, so that the obtained excitation profile can be used to correct any given localization from a new SMLM list. However, this is not strictly necessary. For example, if the user is interested in analysing small regions from a large image of microtubules, he/she can use the entire list of localizations of microtubules to generate the excitation profile. In this way, even though there may be some camera pixels that lack the background information (due to the absence of microtubules within that area), those regions will also be empty in the ROI of interest, and thus unnecessary to correct. In contrast, every analysable small region will have its local correction factor, since those same localizations will have been used to generate the excitation profile. For further details of how the software performs the correction due to non-flat illumination, we refer to Table SSD1, item *xi)*.

4. Visualization: Rendering

Whenever there is a scatter plot of *lateral* vs. *axial* positions in the Visualization panel, the "Render" button will be enabled. When performing a Gaussian rendering, a new figure will pop up, where each localization will be represented with a Gaussian function, with standard deviations in the lateral and axial directions defined by the user (see Figure SSD16a). Also, the pixel size will be determined by the "Magnification" and the "Camera pixel size" (for example, if they are set to 100 and 133 nm, the pixel size in the super-resolution image will be 1.33 nm). When the user presses the "Render" button, a message will suggest selecting with the zoom tool (top left) the ROI to be rendered. Furthermore, there is an option to auto-adjust contrast of the output image (with "imadjust" Matlab function) and an option to use a colormap that reflects the *z*-positions. In Figures SSD16 b)-d) we show some super-resolution images rendered with this tool using the available example data. Finally, we remark that the exported processed data (.csv files) includes headers that allow it to be imported into ThunderStorm, where more rendering options are available.

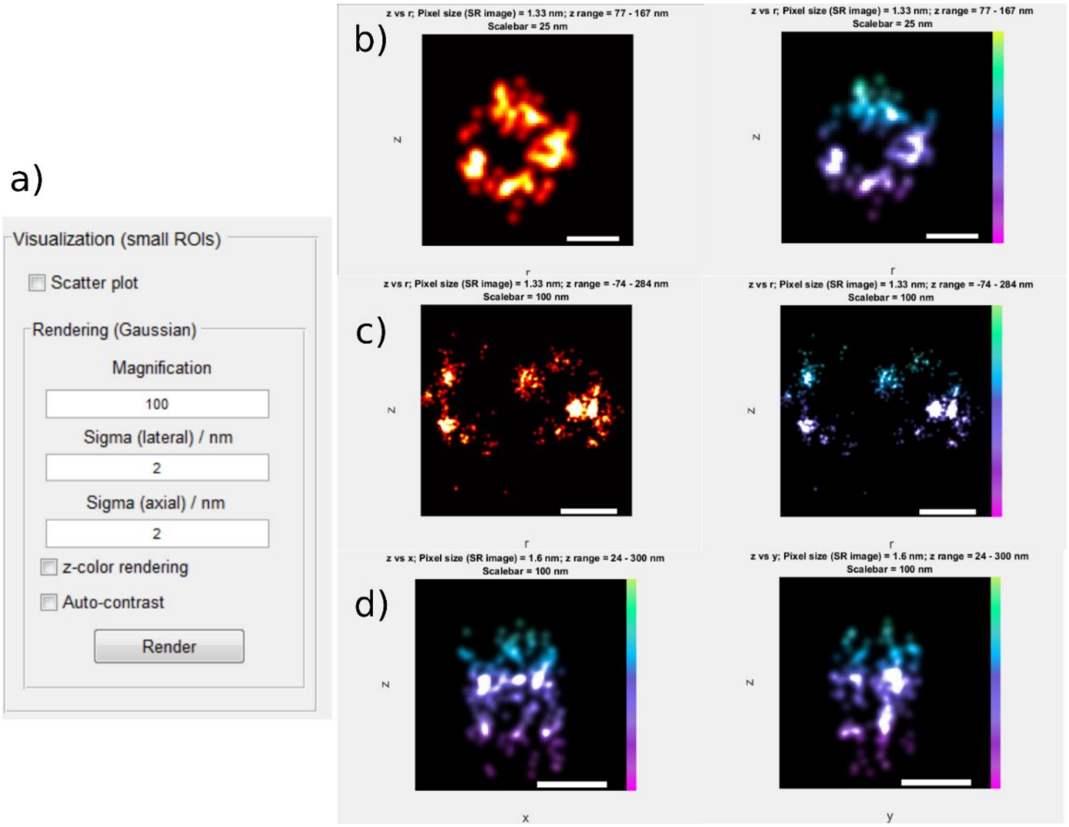

Figure SSD16. a) Rendering (Normalized Gaussian) panel. Magnification, sigma (lateral) and sigma (axial) adjustment. *z*-colour coding and auto-contrast options are displayed. b) to d): Rendered images of b) microtubule's cross-section; c) spectrin ring cross-section (left: no z-colour code; right: z-colour coded); d) side views of single NPC (left: *(x,z)*; right: *(y,z)*).

5. References

[1] Schnitzbauer, J., Strauss, M. T., Schlichthaerle, T., Schueder, F. & Jungmann, R. Super-resolution microscopy with DNA-PAINT. *Nat. Protoc.* **12**, 1198–1228 (2017).

[2] Ovesný, M., Křížek, P., Borkovec, J., Švindrych, Z. & Hagen, G. M. ThunderSTORM: A comprehensive ImageJ plug-in for PALM and STORM data analysis and super-resolution imaging. *Bioinformatics* (2014) doi:10.1093/bioinformatics/btu202.
