# Supplementary figures and images for: Three-dimensional total-internal reflection fluorescence nanoscopy with nanometric axial resolution by photometric localization of single molecules
Source: Nat Commun. 2021 Jan 22;12:517. doi: 10.1038/s41467-020-20863-0 (PMC7822951; doi:10.1038/s41467-020-20863-0)

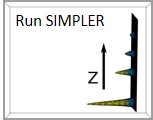

Supplement: Supplementary file 3 — Supplementary Software [file 41467_2020_20863_MOESM3_ESM.zip › SIMPLER Supplementary Software/logo3.jpg]
